# Template Free Anisotropically Grown Gold Nanocluster Based Electrochemical Immunosensor for Ultralow Detection of Cardiac Troponin I

**DOI:** 10.3390/bios12121144

**Published:** 2022-12-07

**Authors:** Sumaya Nisar, Chansi, Ashish Mathur, Tinku Basu, Kshitij RB Singh, Jay Singh

**Affiliations:** 1 Indian Institute of Technology Delhi, Hauz Khas, New Delhi 110016, Delhi, India; 2 Amity Centre for Nanomedicine, Amity University, Noida 201301, Uttar Pradesh, India; 3 Centre for Interdisciplinary Research and Innovation (CIDRI), University of Petroleum and Energy Studies (UPES), Dehradun 248007, Uttarakhan, India; 4 Department of Chemistry, Institute of Science, Banaras Hindu University, Varanasi 221005, Uttar Pradesh, India

**Keywords:** gold nanoclusters (AuNCs), cardiac troponin-I, heart attack management, immunosensors, biosensors

## Abstract

Anisotropic gold nanostructures have fascinated with their exceptional electronic properties, henceforth exploited for the fabrication of electrochemical sensors. However, their synthesis approaches are tedious and often require a growth template. Modern lifestyle has caused an upsurge in the risk of heart attack and requires urgent medical attention. Cardiac troponin I can serve as a biomarker in identification of suspected myocardial infection (heart attack). Hence the present work demonstrates the fabrication of a sensing platform developed by assimilating anisotropic gold nanoclusters (AuNCs) with anti cTnI antibody (acTnI) for the detection of cardiac troponin I (cTnI). The uniqueness and ease of synthesis by a template-free approach provides an extra edge for the fabrication of AuNC coated electrodes. The template-free growth of anisotropic AuNCs onto the indium tin oxide (ITO) glass substrates offers high sensitivity (2.2 × 10^−4^ A ng^−1^ mL cm^−2^) to the developed sensor. The immunosensor was validated by spiking different concentrations of cTnI in artificial serum with negligible interference under optimized conditions. The sensor shows a wide range of detection from 0.06–100 ng/mL with an ultralow detection limit. Thus, it suggests that the template-free immunosensor can potentially be used to screen the traces of cTnI present in blood serum samples, and the AuNCs based platform holds great promise as a transduction matrix, hence it can be exploited for broader sensing applications.

## 1. Introduction

The anisotropic behaviour of gold nanostructures reveals the few excellent advantages over isotropic spherical contour depending on aspect ratio and target application [1]. 1D nanostructures show ballistic electron transport as the dimension of the rod is reduced to a mean free path electron. Thus, it offers a least resistive path for electrons to move, leading to enhanced conductivity. An anisotropic gold nanostructure exhibits exceptional features in sensing attributes including electrochemical and optical properties. The kinetics of the redox reactions is also studied by using anisotropic 1D nanostructures such as nanowires, nanobelts, and gold nanoclusters [2,3]. This led to the exploitation of these nanostructures towards extremely precise sensing with ultra-low detection for diagnostic applications [4], making them an ideal candidate for early sensing of Acute Myocardial Infarction (AMI). Compared to gold nanoparticles, gold nanoclusters (AuNCs) display superior electrochemical sensing for anticancer drugs and cytosine in terms of excellent electrode kinetics and diffusivity [5]. Acute AMI comes under acute coronary syndrome, which is frequently identified as a heart attack. AMI is the critical manifestation of coronary ischemia [6]. It has become a major cause of increased morbidity and mortality rates worldwide [7]. AMI occurs as the consequence of a sudden blockage of blood flow to part of the myocardium (heart muscles). In the United States of America, approximately one million people suffer from a heart attack each year [8]. In the European Union, 50% of the total death rate occurs due to AMI [9]. After heart attack, “Cardiac Biomarkers” or “Enzymes” such as myoglobin, Creatine kinase, Cardiac Troponin I & T are discharged into the blood vessels from the damaged tissue of myocardium [10]. For the diagnosis of AMI, these Cardiac Biomarkers perform a substantial function as they may be detected at one-of-a-kind ranges in blood samples. Cardiac troponin I (cTnI) is considered as the standard biological index for the prognosis of a patient susceptible to heart attack, as it increases specifically during myocardial damage (AMI) [7,11]. Hence, there is an urgent need for the quick monitoring of cTnI for prevention and early treatment of heart attack. Conventional gold standard analytical methods such as enzyme-linked immunosorbent assay (ELISA), radioimmunoassay, and electro-chemiluminescent immune-assay (ECLIA) [12,13] are ideal for the precise detection of cardiac biomarkers, but have a few limitations including high turnaround time, multi-step processing of samples, high cost, less sensitivity, and the requirement for skilled labor. These present a real bottleneck in terms of providing a rapid diagnosis. Currently, electrochemical biosensors are viewed as a potential alternative to the analytical gold standard method for detection of cTnI and other biomarkers with respect to their rapid sensing and a user friendly approach due to its simplicity, enhanced sensitivity, selectivity, low detection limit, less sample volume, cost-effectiveness, and less time consumption qualities [14,15]. The presence of cTnI in serum is sensed through the specific binding of anti-cTnI with cTnI antigen and not with globular protein. cTnI biomarker adopts an extended conformation, as seen in epitope mapping and accurate secondary structure prediction methods, which allows a large sequence of the amino acid to be affirmed by anti-cTnI antibodies (acTnI) [16]. Depending on the levels of cTnI in the human body, the issues may be as follows.

(i) 0–0.4 ng/mL: healthy person
(ii) Between 0.4 and 4 ng mL^−1^: minor heart disorder and unstable angina
(iii) Above 4 ng mL^−1^: risk of major heart attack [16,17].

Due to greater ion conduction, nanomaterial based sensors provide a very low limit of detection (LOD) with excellent sensitivity [18,19,20,21,22]. An electrochemical sensor with horseradish peroxidase (HRP) enzyme is designed from poly(diallyldimethylammonium) reduced graphene oxide, and has a LOD value around 0.024 ng mL for cTnI detection [23]. Bhatnagar et al. have developed graphene based quantum dots (GQD) and electrochemical sensors with poly-amidoamine (PAMAM) nanohybrid modified Au electrodes for cTnI having a sensitivity of 109.23 μA cm^−2^μg^−1^ with a detection limit of 20 fg mL^−1^ [24]. The success rate of commercially available diagnostic biosensors is significantly low. One reason could be their narrow detection range. For instance, at the time of sudden AMI, the level of cTnI may rise above 100 ng/mL, hence a wide detection range is required to detect a healthy state, the risk of a heart attack, and also the susceptibility to a heart attack. Apart from this, other constraints are poor reliability and the complex methodology required to design the platform. To circumvent the above issue the present manuscript describes a novel electrochemical immunosensor built over anisotropic AuNCs grown on indium tin oxide glass substrate (AuNCs/APTMS/ITO) for the broad range detection of cardiac biomarker cTnI. In this experiment, AuNCs were directly grown using cetyltrimethylammonium bromide (CTAB) as a soft template over a APTMS modified indium tin oxide glass electrode. Activated anti-cardiac Troponin I (acTnI) covalently binds with the cysteamine functionalized AuNCs/APTMS/ITO electrode. The present sensor can be distinguished with available techniques in terms of linearity, a broad range of detection (0.06–100 ng/mL), the ease of preparation, and sensitivity (2.2 × 10^−4^ A ng^−1^ mL cm^−2^). The developed immunoelectrode was also additionally used for detection of spiked cTnI in commercially available serum with minimum interference (<15%). The fabricated immunosensor is stable for a month, conforming its translational potential.

## 2. Materials and Methods

### 2.1. Materials

The reagents used for the synthesis of ultra-small AuNCs and sensing platform for Troponin I, artificial serum, and cardiac biomarker were purchased from Sigma–Aldrich (see details in supplementary file). Both hydrochloric acid and methanol were procured from Merck. The solutions were prepared exclusively using deionized water.

### 2.2. Synthesis of Gold Seeds

To 37 mL of deionized water,1 mL of 0.01 M HAuCl_4_ and 0.02 M trisodium citrate was added and stirred. To the resultant solution, an ice-cold solution of 0.01 M NaBH_4_ was mixed slowly in a dropwise manner and stirred. The solution was kept undisturbed for 24 h, filtered and dried. Au nano seeds thus obtained were of 3–5 nm in size.

### 2.3. Preparation of APTMS/ITO Electrodes

The indium tin oxide glass (ITO) substrate of 1.5 cm × 1 cm size was cleaned thoroughly before use and air dried. Then, the ITO substrate was dipped in 10% HCl solution for about 10 min followed by annealing at the temperature ranges of 450–500 °C for 7 h and suddenly cooled to normal room temperature [15,25].

Finally, the ITO substrate was immersed in 30 mM of aminopropyl tri-methoxy silane solution (APTMS) in methanol for about an hour for functionalization. After methanol wash, the ITO substrate was annealed at 100–150 °C for 2 h.

### 2.4. Fabrication of AuNCs (Gold Nanoclusters) Modified ITO Electrode (AuNCs/APTMS/ITO)

The APTMS/ITO electrodes were immersed in gold seed solution and in 1, 12-dodecanedithiol in sequence to obtain a bilayer assembly of seed particles. After each step, the modified electrodes were washed with pure water. The bi-layer seed coated indium tin oxide glass electrode was immersed in 20 mL of 0.2 M cetyltrimethyl ammonium bromide (CTAB) solution, a powerful surfactant, for 5 min. The resultant solution was mixed with 900 µL of 0.01 M HAuCl_4_ and 100 µL of 0⋅1 M ascorbic acid. For 24 h the temperature of the vessel was maintained at 4 °C. After the completion of growth at the specific time, the electrodes were washed 3 times with deionized water to eliminate extra CTAB and other reagents.

### 2.5. Development of BSA/acTnI/Cys/AuNCs/APTMS/ITO Immunoelectrode

The AuNCs/APTMS/ITO was improved further to develop as immunoelectrodes. The amine group was loaded on the electrode surface by dipping it in a 0.4 m aqueous solution of Cystaemine (Cys) for 24 h. After the deposition of Cys, electrodes were washed with water and dried. 50 µg/mL of anti-cTnI antibody was used for electrode fabrication. Activation of acTnI antibodies was done with 0.2 M of 3-dimethylaminopropyl-N′-ethylcarbodiimide hydrochloride & 0.05 M NHS (N-hydroxysuccinimide) solution for about 1–2 h. Approximately 10 µL of activated-antibody solution was spurted on Cys/AuNCs/APTMS/ITO electrode and then incubated for 24 h at 4 °C. During the incubation period, a new amide bond is formed between the antibody and cysteamine. The non-specific binding of fabricated acTnI/Cys/AuNCs/APTMS/ITO immunoelectrode was prevented by adding 1 mg/mL commercially available bovine serum albumin. These fabricated BSA/acTnI/Cys/AuNCs/APTMS/ITO immunoelectrodes were used for cTnI detection using cyclic voltammetry. After each deposition, the fabricated immunoelectrode was washed with phosphate buffer saline to maintain pH.

### 2.6. Optimization of Operational Parameters of BSA/acTnI/Cys/AuNCs/APTMS/ITO Immuno-Electrodes

The processing and operational parameters of the fabricated immuno-electrode were optimized by changing the concentration of acTnI (30 µg/mL to 60 µg/mL), cysteamine (2 mM to 5 mM), the incubation time (10 m to 40 m), and operational pH (6.5 to 7.8), respectively. The anodic (oxidation) peak current of cyclic voltammetry in phosphate buffer saline containing 5 mM [Fe(CN)_6_]^3−/4−^ at each case is noted. The optimum process parameters thus finalized were 50 µg/mL of acTnI, 4 mM of cysteamine of concentration, 20 min of incubation time, and 7.4 pH. (Supplementary Information Figures S3–S6). Interaction of the immuno-electrode with cTnI enhances the anodic peak current; the highest anodic peak current observed at each parameter variation is recorded as the optimum response.

### 2.7. Detection of cTnI Using Developed BSA/acTnI/Cys/AuNCs/APTMS/ITO Immunoelectrode

The cyclic voltammetry analysis of BSA/acTnI/Cys/AuNCs/APTMS/ITO electrodes with varying concentrations of cTnI were studied in phosphate buffer saline solution using 5 mM of [Fe(CN)_6_]^3−/4−^ as redox probe under suitable experimental conditions. The CV (cyclic voltammetry) measurement was carried using a 3-electrode system with the developed immunoelectrode as working electrode, Pt wire as counter electrode & saturated Ag/AgCl as the reference electrode in the aforesaid PBS buffer at 25 °C. The experiments were repeated three times to get a concordant value.

### 2.8. Determination of cTnI Using Developed BSA/acTnI/Cys/AuNCs/APTMS/ITO Immunoelectrodes in Spiked Serum Sample

Commercially available serum was taken as control and, further, it was spiked with known concentrations (covering 70 to 0.5 ng mL^−1^) of cTnI for evaluation of immunosensor response to the pooled serum.

### 2.9. Stability Studies

The stability of the AuNCs/APTMS/ITO electrode along with the immunoelectrode stability was studied. Sixty AuNCs/APTMS/ITO electrode and BSA/acTnI/Cys/AuNCs/APTMS/ITO immune electrodes were developed and maintained at 4 °C. Their cyclic voltammetry responses were investigated over a period of 30 days.

## 3. Characterization

Cyclic voltammetry studies have been executed with Autolab Potentiostat/Galvanostat Model AUT83945 (PGSTAT302N) with a 3-electrode system. A fabricated immunosensor electrode BSA/acTnI/Cys/AuNCs/APTMS/ITO was used as a working electrode with platinum wire (Pt) and saturated Ag/AgCl as counter and reference electrode in PBS solution containing 5 mM [Fe(CN)_6_]^3−/4−^ as mediator. The surface morphological studies and topology were examined by using FE-SEM: MIRA II LMH and AFM-NDMT. An elemental composition was carried out with the support of Energy Dispersive X-ray diffraction (EDAX) (INCA PentaFET3). Bonding and absorption phenomena were evaluated from an FTIR spectrometer and UV-visible spectra were recorded using a Hitachi U3300 spectrophotometer.

## 4. Result and Discussion

### 4.1. Structural and Morphological Analysis

#### 4.1.1. SEM and AFM Analysis

**Figure 1:**
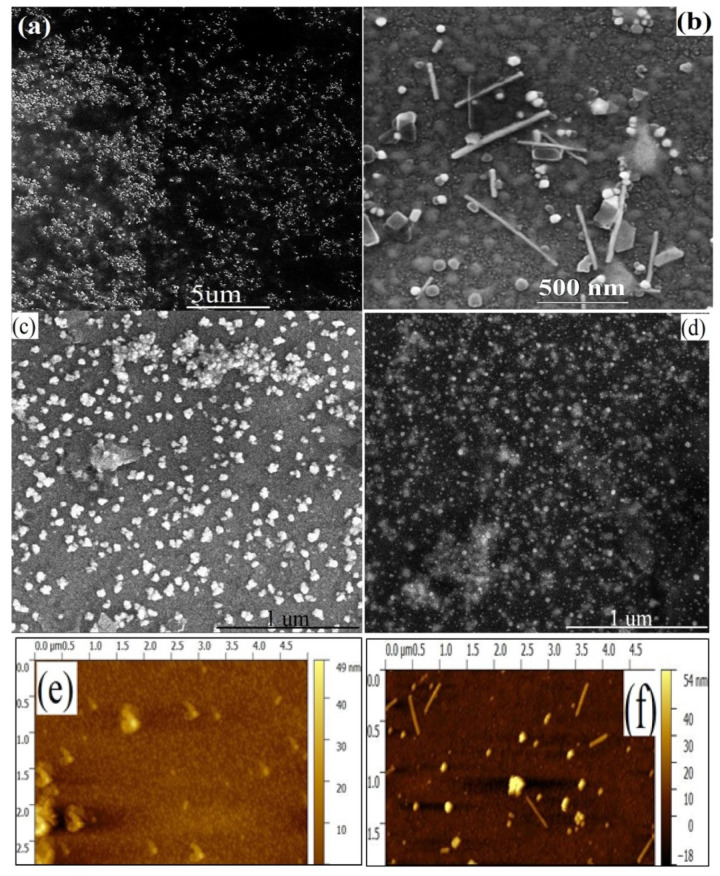
FESEM images of surface morphology of (**a**) Au bilayer (**b**) AuNCs/APTMS/ITO in (**c**) acTnI/Cys/AuNCs/APTMS/ITO and (**d**) BSA/acTnI/Cys/AuNCs/APTMS/ITO at a magnification of 10 kx, 100 kx and 60 kx respectively AFM image of (**e**) Au Bilayer (**f**) AuNCs/APTMS/ITO AFM images of surface topography for 2.5 µm × 5 µm area and 1.5 µm × 5 µm of developed electrode, respectively.

AuNCs were allowed to grow directly on APTMS/ITO electrodes. FESEM image analysis is an important tool to visualize the surface morphology in the nanometer scale. Uneven distribution and scattered arrangement of the bilayer assembly of Au seeds over APTMS/ITO were captured by FE-SEM image analysis (Figure 1a). A dense population of Au seed of bilayer assembly is observed in the FE-SEM image (Figure 1a) onto the APTMS/ITO surface which acts as the nucleation for growth of nanoclusters. A cylinder-shaped micelle is formed on the external face of the electrode due to the influence of CTAB on the growth of nanoclusters.

The seed particles will attach either at the end or onto the surface of the micelle [26]. Due to the presence of the cylindrical micelle, facet-controlled growth occurs. After 24 h, nanoclusters are fully grown on the APTMS/ITO surface along with gold nanopellets of different shapes and sizes which are visible in the SEM and AFM image (Figure 1b,e,f). The length of the rod varies from 60 nm to 600 nm with an average aspect ratio of 20:1. FESEM reveals that the diameter of the rod is constrained to 10 to 30 nm. AFM was also used to measure the thickness of the developed electrode at each stage. The height profile indicates that the bilayer seed assembly and the developed electrode are 60 nm and 350 nm thick, respectively. Figure 1c shows the FE-SEM image of immobilized acTnI antibodies onto the Cys/AuNCs/APTMS/ITO electrode. The formation of small clusters shows the presence of immobilized antibodies. The FE-SEM image of the developed BSA/acTnI/Cys/AuNCs/APTMS/ITO immunoelectrode [27] is shown in Figure 1d.

#### 4.1.2. UV-Visible Spectroscopy

**Figure 2:**
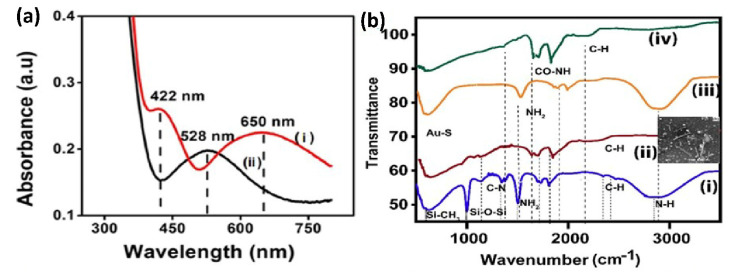
(**a**) UV-Visible Spectra of (i) Au Bilayer with absorption maxima at 528 nm (ii) AuNCs/APTMS/ITO with absorption bands at 422 nm and 650 nm. (**b**) FTIR Spectra of (i) APTMS/ITO electrode (ii) Cys/AuNCs/APTMS/ITO (iii) acTnI/Cys/AuNCs/APTMS/ITO electrodes obtained from 600 cm^−1^ to 5000 cm^−1^.

The UV-visible spectra of the Au bilayer and for AuNCs on APTMS/ITO electrodes was shown in Figure 2a. The bilayer assembly exhibits a peak at 528 nm due to surface plasmon of spherical gold nanoparticles as represented by graph (a) curve (i) in Figure 2. On the contrary, anisotropic AuNCs and nanopellets of different shapes exhibit two absorption bands due to the plasmon resonance corresponding to the transverse (422 nm) and longitudinal dimensions (650 nm) of anisotropic nanostructures (curve ii in Figure 2a). The blue shift of TSPR and the red shift of LSPR show the presence of long nanoclusters with a small diameter. Therefore, UV-Visible analysis confirms the formation of AuNCs on the APTMS/ITO surface.

#### 4.1.3. IR Spectroscopy

Figure 2b displays IR Spectra of (i) APTMS/ITO electrode (ii) AuNCs/APTMS/ITO (iii) Cys/AuNCs/APTMS/ITO electrodes and (iv) acTnI/Cys/AuNCs/APTMS/ITO. The IR spectra of the APTMS functionalized ITO surface shows absorption bands at 648 and 1016 cm^−1^, which corresponds to the symmetric and antisymmetric stretching frequency of Si-CH_3_ and Si-O-Si respectively and confirms the bond formation between silane and the ITO surface [28]. Several other peaks which are attributed to C-N stretching are at 1404 cm^−1^, C-H at 2843 cm^−1^, and N-H stretch at 3310 cm^−1^, respectively. The presence of NH_2_ groups at the silanized ITO surface was confirmed by NH_2_ bending deformation peak at 1620 cm^−1^ (curve i). AuNCs/APTMSITO (curve ii), which displays the growth of anisotropic nanorods over the functionalized ITO surface, do not witness a marked difference in the peak, however the intensity of the peaks is reduced due to the entire surface coverage (as shown in the FESEM image attached). Further, as the surface is modified with cysteamine (curve iii), we observe a broad amine bending peak at around 1641 cm^−1^ attributed to the fact that both APTMS and cysteamine provide amine functionalities. Though the amine bending vibrations are present, the S-H band (2550 cm^−1^ seen in pure cysteamine) is not noticed in the spectra of cysteamine modified AuNCs surface due to Au-S interaction [21]. In addition, a peak which is spotted at 638 cm^−1^ confirms that the AuNCs surface is thiolated by the -SH group of cysteamine [28,29]. Curve (iv) displays the immobilization of acTnI over the cysteamine modified surface as the amide I peak is observed at 1661 cm^−1^ formed by the covalent reaction between the COOH group (free carboxylic peak observed at 1710 cm^−1^) of the activated antibody and amine group from the cysteamine modified surface (curve iii) [29,30].

The EDAX study reveals the presence of pure Au^0^ on the developed electrode. Figure S1 (Supplementary) ((i) (ii)), shows the spectra of the Au seed layer and the AuNCs/APTMS/ITO electrode, respectively. From seed assembly to the developed AuNCs electrode, the increase in weight % and atomic % of Au^0^ is around 31.52% and 19.89%, respectively, further confirming the enhanced density of Au over the AuNCs/APTMS/ITO electrode.

### 4.2. Electrochemical Characterization of the Developed Electrodes

#### 4.2.1. Cyclic Voltammetry Study

**Figure 3:**
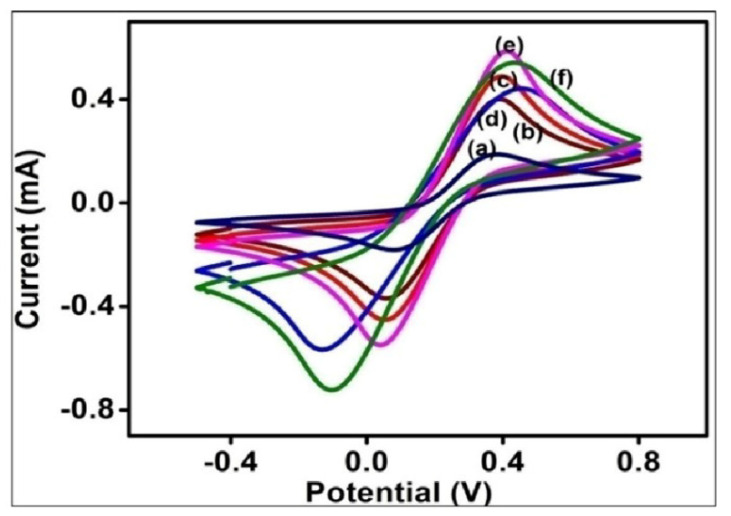
(**a**) Bare ITO (**b**) Au bilayer (**c**) AuNCs/APTMS/ITO (**d**) Cys/AuNCs/APTMS/ITO (**e**) acTnI/Cys/AuNCs/APTMS/ITO (**f**) BSA/acTnI/Cys/AuNCs/APTMS/ITO at a scan rate of 100 mV/S within a potential −0.4 V to 0.8 V in PBS buffer containing [Fe(CN)_6_]^3−/4−^.

Cyclic voltammetry (Figure 3) was carried out at a scan rate of 100 mVs^−1^ from −0.4 V to 0.8 V. Excellent redox signals have been reported from all the electrodes regardless of the modifications [Figure 3]. The bare ITO shows a peak current intensity of 1.875 × 10^−4^ A due to its electrically conductive nature. On grafting Au seeds (bilayer assembly) on to the APTMS/ITO, the magnitude peak current is increased to 4.005 × 10^−4^ A due to the high conductivity of Au nanoparticles. The growth of anisotropic AuNCs structures onto the APTMS/ITO electrode records another rise in peak current density up to 4.889 × 10^−4^ A in the AuNCs/APTMS/ITO electrode. The ballistic electron transport of nanoclusters allows higher conductivity, thus increasing the sensitivity of the developed sensor. The cysteamine functionalized (Cys/AuNCs/APTMS/ITO) electrode displays a plummet in the peak current magnitude (4.41711 × 10^−4^ A) and a slight shift in potential towards higher values confirms the presence of insulating cysteamine. It is noted that the enhancement of current density from the gold seed/APTMS/ITO to the AuNCs/APTMS/ITO electrode is not prominently increased, which may be ascribed by the presence of the organic shape-directing agent CTAB. The binding of acTnI on the Cys/AuNCs/APTMS/ITO electrode increases the peak current to 5.867 × 10^−4^ A. This can be due to the presence of polar positively charged amine functionalities onto the activated antibody [26]. To block any non-specific site onto the developed immunoelectrode (acTnI/Cys/AuNCs/APTMS/ITO), BSA was used, which causes a diminution in peak current intensity up to 5.404 × 10^−4^ A. The presence of organic groups in BSA protein prevents the electron movement among the electrolyte and the working electrode, and hence it affects the quasi-reversible characteristics of the cell. As a result, the current intensity drops down and a potential shift towards a higher value is also observed. The peak current ratio (Ipa/Ipc) is almost close to 1 in all the cases confirming the reversible behavior of the electrodes irrespective of the extent of modification.

**Figure 4:**
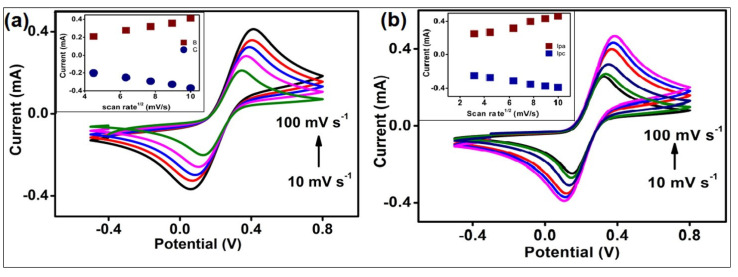
Cyclic voltammetry of (**a**) AuNCs/APTMS/ITO and (**b**) BSA/acTnI/Cys/AuNCs/APTMS/ITO and inset of (**a**,**b**) show the calibration plot of Ipa and Ipc with a scan rate of 10–100 mV/s.

The cyclic voltammetry of AuNCs/APTMS/ITO and the BSA/acTnI/Cys/AuNCs/APTMS/ITO immunoelectrode (Figure 4) was studied with a 10–100 mV s^−1^ scan rate. Since the value of both cathodic (Ipc) and anodic (Ipa) peak currents rises progressively with the square root of the scan rate, there is more possibility for a diffusion-controlled process. The slopes and intercepts for AuNCs/APTMS/ITO and BSA/acTnI/Cys/AuNCs/APTMS/ITO are given by the following equations.

AuNCs/APTMS/ITO:

Ipa = 0.054 (mA) + 0.035 mA^2^ mV s^−1^ {scan rate (mV s)} ^1/2^ R^2^ = 0.96 (1)

Ipc = −0.029 (mA) − 0.06 mA^2^ mV s^−1^ {scan rate (mV s)} ^1/2^ R^2^ = 0.99 (2)

BSA/acTnI/Cys/AuNCs/APTMS/ITO:

Ipa = 0.033 (mA) + 0.032 mA^2^ mV s^−1^ {scan rate (mV s)} ^1/2^ R^2^ = 0.99 (3)

Ipc = −0.017 (mA) − 0.021 mA^2^ mV s^−1^ {scan rate (mV s)} ^1/2^ R^2^ = 0.99 (4)

Both peak potentials (Epa & Epc) and the difference between anodic and cathodic peak potential (ΔE) rises with the rise in scan rate. The linear regression coefficient value is 0.97, which shows a facile interfacial charge transfer mechanism between 10–100 mV s^−1^ scan rate. The BSA/acTnI/AuNCs/APTMS/ITO electrode offers enough possibility to electrons to shuffle among the antibodies and the electrode.

For redox particles, the diffusion coefficient value (D) from the electrolyte solution to the AuNCs/APTMS/ITO electrode and BSA/acTnI/Cys/AuNCs/APTMS/ITO immunoelectrode were determined from the Randles–Sevcik equation [31], where Ip = peak current intensity of the electrode, n = number of electrons involved, A = surface area of the electrode (0.28 cm^2^), D = diffusion coefficient, C = concentration of the redox species (5 mM [Fe(CN)_6_]^3−/4−^), and ʋ = scan rate (100 mV s^−1^).

Ip = (2.69 × 10^5^)n^3/2^AD^1/2^Cʋ^1/2^

The D value is found as 1.25 × 10^−3^ cm^2^ s^−1^ for AuNCs/APTMS/ITO electrodes and 1.19 × 10^−3^ cm^2^ s^−1^ for BSA/acTnI/Cys/AuNCs/APTMS/ITO immunoelectrode. The value of D is higher for AuNCs/APTMS/ITO electrode.

The presence of cTnI in commercial serum is sensed through the specific binding of anti-cTnI with cTnI antigen and not with globular protein, hence the surface concentration of the AuNCs/APTMS/ITO electrode and the BSA/acTnI/Cys/AuNCs/APTMS/ITO immunoelectrode were calculated using a cyclic voltammetry plot with the help of the Brown–Anson model [32], where *n* = no. of electrons transferred, *F*—Faraday constant, *g*—surface concentration of the respective electrode, *A*—surface area of the electrode (0.28 cm^2^), *ʋ* = scan rate (100 mV s^−1^), *R* = gas constant, and *T* = room temperature.

$$Ip=\frac{n^{2}F^{2}gAʋ}{4RT}$$

The concentration of the BSA/acTnI/Cys/AuNCs/APTMS/ITO immunoelectrode (19.70 × 10^−4^ mol cm^−2^) electrode surface is more than AuNCs/APTMS/ITO electrode (17.9 × 10^−4^ mol cm^−2^). The *Ks* value (heterogeneous electron—transfer rate constant) for the AuNCs/APTMS/ITO electrode & BSA/acTnI/Cys/AuNCs/APTMS/ITO immunoelectrode were determined from the following Lavrion model [31,33], where m value gives peak-to-peak separation.

$$Ks=\frac{mnFʋ}{RT}$$

**Figure 5:**
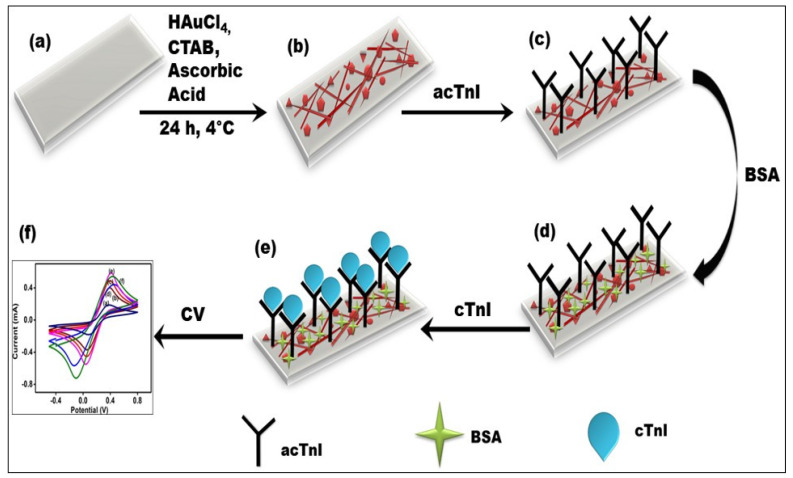
Schematic illustration of the fabrication steps of BSA/acTnI/Cys/AuNCs/APTMS/ITO immunoelectrodes (**a**) Bare ITO (**b**) AuNCs/APTMS/ITO (**c**) acTnI/Cys/AuNCs/APTMS/ITO (**d**) BSA/acTnI/Cys/AuNCs/APTMS/ITO (**e**) cTnI/BSA/acTnI/Cys/AuNCs/APTMS/ITO (**f**) cyclic voltammetry graph of surface modifications.

The *Ks* value was found to be 1.36 s^−1^ for AuNCs/APTMS/ITO, which was higher than the 1.17 s^−1^ electrode for BSA/acTnI/Cys/AuNCs/APTMS/ITO immunoelectrode. This confirms the electron shuffling between the immobilized antibodies and electrodes. Figure 5 shows the representation diagram of the fabrication steps of immunoelectrodes.

#### 4.2.2. Optimization of Variables

The effect of pH, antibody and cysteamine concentration, interaction and working potential of the fabricated immunoelectrode, and the incubation time for the antibody and antigen (acTnI and cTnI), were examined by CV in PBS at a scan rate of 30 mV. The maximum response between the antibody and antigen (cTnI) interaction was found in optimum value (50 µg/mL) of acTnI with an incubation time of 35 min. The maximum peak current for redox potential was obtained at pH 7.7 with a shift in Epa towards the lowest values. A further increase in pH decreases the peak current. Thus, the pH 7.4 is designated as the optimized pH for further investigation (Figures S3–S6, Supplementary File).

#### 4.2.3. Immunosensor—Response Studies

**Figure 6:**
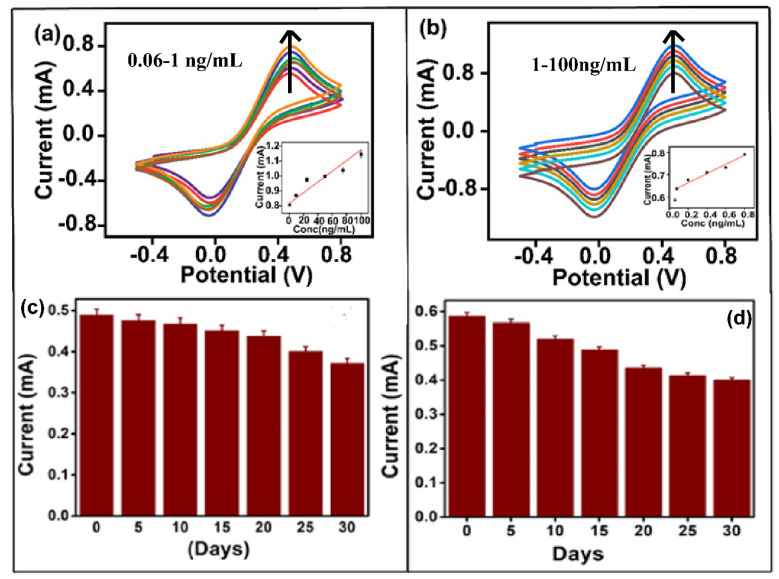
(**a**) Cyclic voltametric response of fabricated immunosensor (at concentration 0.06–1 ng/mL) Inset: Calibration between the magnitude of anodic peak current (mA) and concentration (ng/mL) (**b**) Cyclic voltametric response of fabricated immunosensor (at concentration 1–100 ng/mL) Inset: Calibration between the magnitude of anodic peak current (mA) and concentration (ng/mL), (**c**) shelf-life study of the AuNCs/APTMS/ITO and (**d**) BSA/acTnI/Cys/AuNCs/ITO immunoelectrode for a period of 30 days.

The efficacy of the BSA/acTnI/Cys/AuNCs/APTMS/ITO immunoelectrode was examined with the help of cyclic voltammetry in PBS with [Fe(CN)_6_]^−3/−4^ from −0.4–0.8 V at a scan rate of 100 mV s^−1^ as a function of cTnI concentration (0.06–100 ng mL^−1^). Figure 6a,b displays the cyclic voltametric response curve along with the calibration plot (inset) of the developed immunosensor. A calibration curve was plotted against a measure of anodic electric current vs. concentration of cTnI. The peak current magnitude increases with rise in concentration of cTnI against the BSA/acTnI/Cys/AuNCs/ITO immunoelectrode. This is attributed due to the enhanced electron transfer onto the BSA/acTnI/Cys/AuNCs/APTMS/ITO electrode after the adsorption of cTnI. Two calibration curves are obtained: one at a low concentration of cTnI (0.06 to 1.0 ng/mL) and the second for a higher concentration of cTnI (1 to 100 ng/mL). Due to the wide range, two plots are obtained. The cyclic calibration plots (Figure 6) show a linear relationship with an average regression coefficient (R^2^) of the plots of 0.9421, which indicates their linearity. The regression equations obtained by plotting the anodic peak current vs. concentration is shown below, where, ‘I’ is anodic peak current and C is concentration.

I = 0.84 + 3.3 × 10^−3^C

I = 0.62 + 0.23C

From the calibration curve, the sensitivity and detection limit was calculated as 2.2 × 10^−4^ A ng^−1^ mL cm^−2^ and limit of detection: 0.043 ng/mL. With the help of a Line weaver–Burke plot, the average association constant (*Ka*) was calculated as 3.025 × 10^−2^ M. The *Ka* value shows a high affinity and strong bonding of cTnI with the BSA/acTnI/Cys/AuNCs/APTMS/ITO immunoelectrode surface due to a favourable rotation of acTnI on the improved surface of the ITO electrode. It is noteworthy that the conformational variations in immunoglobin will influence the cTnI interaction [16]. The remarkability of the developed cTnI immunosensor is its width (0.06–100 ng/mL). However, three key points can be ascertained: (i) the high aspect ratio of nanoclusters randomly scattered over the APTMS/ITO electrode, thus providing a high surface area with electron confinement. The existence of nanoclusters increases the number of active surface sites, thus enhancing the chemical reactivity and surface bonding characteristics of AuNCs for the immobilization of antibodies. The size and shape-dependent electrochemical properties of anisotropic nanosystems arise due to (ii) spatial confinement and the restricted motion of electrons, holes, excitons, plasmons, and phonos. (iii) The favorable immobilization of the acTnI—amine group of Cys forms a covalent bond with the acTnI—Fc part to project the Fab part (the Fab part determines specificity and promotes antigen binding) of acTnI towards the antigen to have optimum interaction, and the direct linkage of acTnI onto the electrode-acTnI is attached through Cys moiety, which leads to the direct connection of acTnI on the electrode. Table 1 presents the comparison data of the aforementioned immunosensor with some current sensors [23,24,32,34,35].

#### 4.2.4. Stability and Interference Study of Immunoelectrode

The stability of AuNCs/APTMS/ITO electrodes was compared with BSA/acTnI/Cys/AuNCs/APTMS/ITO immunoelectrodes. Figure 6c,d shows the shelf life study of the developed AuNCs/APTMS/ITO and the BSA/acTnI/Cys/AuNCs/APTMS/ITO immunoelectrode. AuNCs/APTMS/ITO electrodes show good stability up to 25 days. A total of 4.2% of signal variation (after 10 days) and 18% of assessment (after 25 days) is observed. The developed BSA/acTnI/Cys/AuNCs/APTMS/ITO immunoelectrodes show excellent stability for up to 10 days. A signal variation of 11.428% after the 10 day assessment period and 31.81% signal after the 30 day assessment period was observed. The AuNCs/APTMS/ITO electrode is more stable than the BSA/acTnI/Cys/AuNCs/APTMS/ITO immunoelectrodes. This is observed due to the low stability of the antibody. Antibodies are sensitive to pH, variation in temperature, and for a few preservatives, eg. sucrose and glycerol, for their activity [36]. Thus, critical storage conditions play an important role to prevent their aggregation. An interference study was performed for the identification of cTnI at a concentration of 25 ng/mL in the presence of a similar concentration of the isomeric form of cardiac troponin (cTnT) and BSA, respectively. It was observed that the percentage interference by BSA (6%, RSD: 3.26%) and cTnT (14%, RSD: 6.5%) was very much within the permissible limit (<15%) (Supplementary Information, Figure S7).

#### 4.2.5. Measurement of cTnI in Spiked Serum Samples

**Figure 7:**
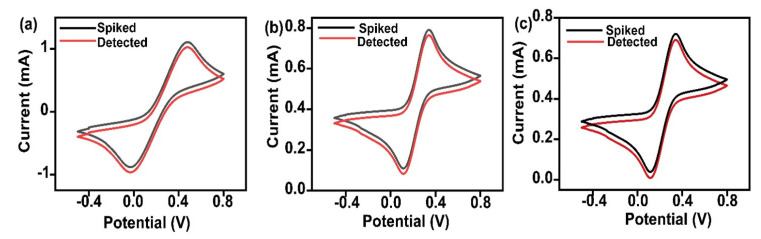
Cyclic voltametric response of the fabricated immunosensor towards the serum samples spiked with various concentrations of cTnI.

Commercial serum samples spiked with specific cTnI at various concentrations were examined by cyclic voltammetry at a scan rate of 100 mV from −0.4 V to +0.8 V. The cyclic voltammetry curves for the concentration spiked and detected concentration was given in Figure 7a–c. Table 2 provides information about percentage error and relative standard deviation. The error was in the range of ±15%. Replacing the polyclonal antibody with the monoclonal one will result in a much lesser percentage error. To estimate the reproducibility and precision of the immunosensor, experiments are conducted in duplicate. This work explored the capacity of developed immunoelectrodes in detecting cTnI in serum without any interferences and specificity.

## 5. Conclusions

A label-free electrochemical immunosensor designed on randomly grown Au Nanoclusters over ITO has been developed to detect cTnI for AMI diagnosis. AuNCs were directly grown onto the APTMS/ITO electrode by means of CTAB, which is a soft template, which hence enables the fabrication of a 1D nanostructure-based electrode. The developed BSA/acTnI/Cys/AuNCs/APTMS/ITO immunosensor exhibits a wide detection range. Two linear detection ranges were obtained, the first for a low concentration of cTnI (0.06 to 1.0 ng/mL) and the second for a higher concentration of cTnI (1 to 100 ng/mL). The developed immunosensor shows a detection limit of around 0.043 ng/mL, sensitivity of 2.2 × 10^−4^ A ng^−1^ mL cm^−2^ with high association constant (Ka) of 3.025 × 10^−2^ M. This novel immunosensor has excellent capacity for the detection of cTnI in spiked serum samples. It provides outstanding sensitivity, wide linear range, and very low LOD with a maximum association constant value in a simple, direct, and label-free environment. These excellent characteristics of the immunosensor are due to the development of a tunable and highly conductive AuNCs/APTMS/ITO base for immunoelectrode development. The proposed AuNCs/ITO electrode can be used as a model for any biological system owing to its high tunability and high electrochemical sensitivity. In any bio-affinity based sensor, sensor changes are less due to the biological interactions occurring. Therefore, it is required to construct a very sensitive and tunable sensor substrate. Efforts are being made to further ease the sensor fabrication process of this device.

## Figures and Tables

**Table 1:** Comparison of developed immunosensor with the available methods in the literature.

S.No.	Matrix	Detection Method	Detection Range	LOD	Sensitivity	Ref.
1	cTnI antibody conjugated nanohybrid modified gold electrode	Electrochemical	10^−6^–10 ng/mL	20 fg/mL	109.23 μA cm^−2^ μg^−1^	[31]
2	acTnI/PDDA/RGO nanocomposite	Electrochemical	0.1–10 ng/mL	0.024 ng/mL		[21]
3	acTnI/(2-ABA) functionalized graphene	Electrochemical	0.01–1 ng/mL	0.01 ng/mL		[32]
4	acTnI/GNPs Matrix/SPE	Electrochemical	0.2–12.5 ng/mL	0.2 ng/mL		[25]
5	acTnI/GNP/ITO	Electrochemical	1 to 100 ng/mL	1 ng/mL		[33]
6	anti-cTnI/afGQDs	FRET	0.001 to 1000 ng/mL	0.192 pg/mL		[22]
7	anti-cTnI/CNFs	Electrochemical	0.25–100 ng/mL	0.2 ng/mL		[34]
8	acTnI/AuNCs/ITO	Electrochemical	0.06–100 ng/mL	0.043 ng/mL	2.2 × 10^−4^ A ng^−1^ mL cm^−2^	Present work

**Table 2:** Detection of cTnI in Serum Samples.

Sample no.	Actual Spiked Concentration (ng/mL)	Experimental Concentration (ng/mL)	% Error	Relative Standard Deviation
1	70	52	25%	6.9%
2	0.8	0.85	6.25%	5.16%
3	0.5	0.43	14%	5.34%

## Data Availability

Data is contained within the article or Supplementary Materials.

## Supplementary Materials

The following supporting information can be downloaded at: https://www.mdpi.com/article/10.3390/bios12121144/s1, Figure S1: EDAX Analysis, Figure S2: EIS spectra of AuNCs/APTMS/ITO electrodes, Figure S3–S6: Optimization of fabricated immunoelectrode: antibody concentration (acTnI), cysteamine concentration, the incubation time for antibody (acTnI) and antigen (cTnI) interaction, and operational pH, and Figure S7: interference study of the proposed immuno-electrode.
