# Supplementary material for: Template Free Anisotropically Grown Gold Nanocluster Based Electrochemical Immunosensor for Ultralow Detection of Cardiac Troponin I
Source: Biosensors (Basel). 2022 Dec 7;12(12):1144. doi: 10.3390/bios12121144 (PMC9775497; doi:10.3390/bios12121144)
Supplement: Supplementary file 1 [file biosensors-12-01144-s001.zip › biosensors-2005080-supplementary.pdf]

Supplementary Information

Template Free Anisotropically Grown Gold Nanocluster Based Electrochemical Immunosensor for Ultralow Detection of Cardiac Troponin I

Sumaya Nisar [1], Chansi [2], Ashish Mathur [3], Tinku Basu [2,*], Kshitij RB Singh [4] and Jay Singh [4,*]

Material and regents

Aurochloric acid ($HAuCl_4$), trisodium citrate, sodium borohydride ($NaBH_4$), 3-Aminopropyl tri-methoxysilane (APTMS), ascorbic acid, 1,12-dodecanedithiol, cetyltrimethyl ammonium bromide (CTAB), (3-Dimethylaminopropyl)-N′-ethylcarbodiimide hydrochloride (EDC),N-Hydroxysuccinimide (NHS), Cystaemine (Cys), cardiac Troponin-I (TNNI1, cTnI), anti- cardiac Troponin-I (AV42118, anti-TNNI1 produced in rabbit, acTnI) were purchased from Sigma–Aldrich. Hydrochloric acid (HCl) and Methanol were procured from Merck and Co, USA. Deionized water (18 MΩ cm) was used for the preparation of solutions.

S1 Energy Dispersive X-ray diffraction (EDAX) analysis

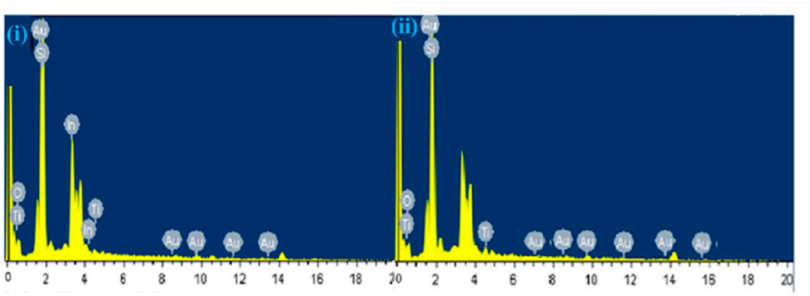

Figure S1. EDAX Analysis of (i) Au seed layer and (ii) AuNCs/APTMS/ITO showing high intensity peaks of elemental Au.

S2. EIS spectra of AuNC/APTMS/ITO electrodes

EIS for Au bilayer APTMS/ITO and AuNC/APTMS/ITO respectively was performed on in PBS, 50 mm, pH 7.5, 0.9% (w/v) NaCl containing 5 mm $[Fe(CN)_6]^{3-/4-}$ as a redox within AC frequency varying from 100 Hz to 100 Khz and Nyquist plot was made as seen **Figure S2**. It can be seen that the R_{CT} value of 1.31 kohms for AuNC/APTMS/ITO (curve b) is much lower than Au bilayer/APTMS/ITO of 2.89 kohms (curve a) proves that AuNC/APTMS/ITO has improved ion conduction. The result is in agreement with CV results.

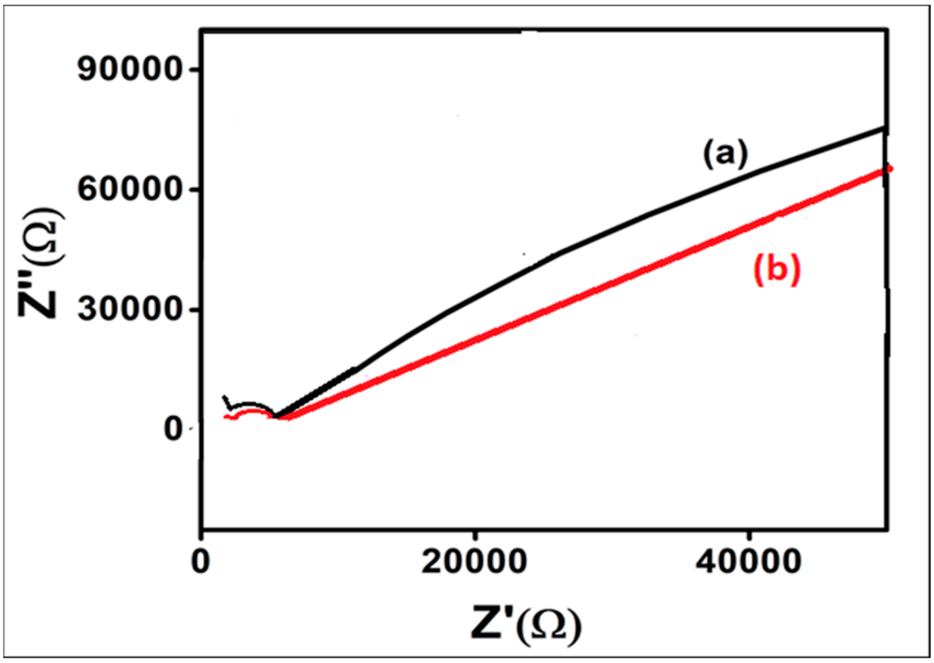

Figure S2. Nyquist plot of (a) Au seeds on APTMS/ITO (b) AuNR/APTMS/ITO electrode in PBS, 50 mM, pH 7.5, 0.9% (w/v) NaCl containing 5 mM $[Fe (CN)_6]^{3-/4-}$ as a redox at AC frequency 100 Hz to 100 KHz.

S3. Optimization of Cysteamine concentration.

The concentration of cysteamine was varied from 2mM to 5mM, for the fabrication of immuno-electrode. Since, cysteamine is a insulating biomolecule it will cause a decrease in anodic peak current on self-assembling over AuNC/APTMS/ITO electrode. Maximum reduction in current was observed at 4mM concentration, after which no significant fall in current was noticed confirming no further incorporation of cysteamine (**Figure S3**) is required. Hence, 4 mM of cysteamine was used as optimized concentration for the entire experiment.

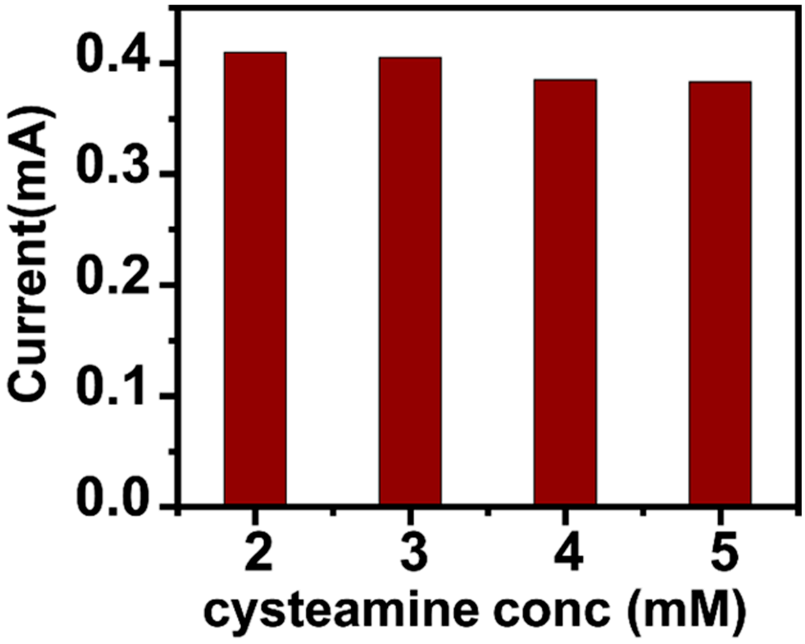

Figure S3. Variation of cysteamine concentration (from 2mM to 5mM) for fabrication of immuno-electrode (BSA/acTnI/Cys/AuNR/APTMS/ITO).

S4. Optimization of acTnI concentration

The concentration of acTnI was varied from 30 µg/mL to 60µg/mL. The optimum loading of acTnI (50µg/mL) was considered by the maximum anodic peak current (0.54 mA) obtained during interaction of immuno-electrode acTnI/Cys/AuNC/APTMS/ITO with cTnI and the lower detection limit (0.0001ng/mL) (**Figure S4**). With very high concentration of antibody, steric hindrance appears which will compromise the detection range. Hence, the loading of acTnI was kept as 50µg/mL for fabricating immuno-electrode for ultra low sensing of cardiac marker Troponin I (cTnI).

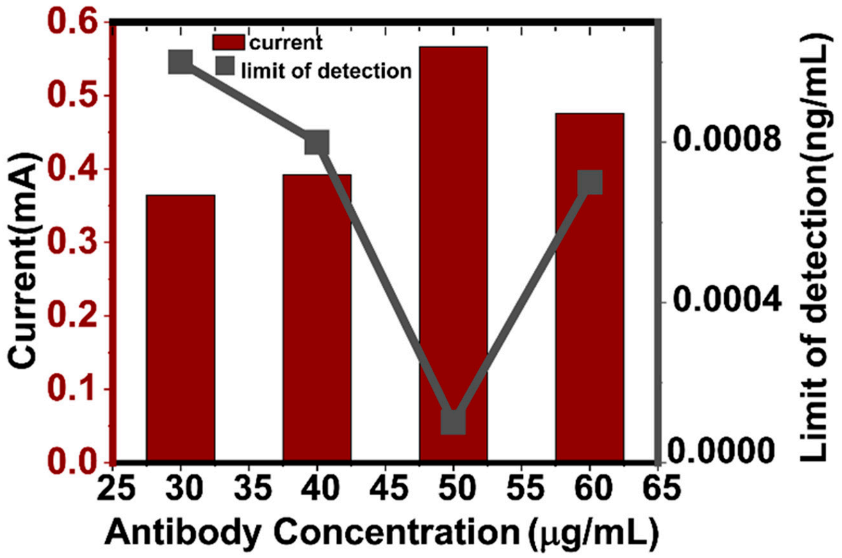

Figure S4. Effect of antibody (acTnI) concentration (variation 30 -60µg/ML-1) on the response of immuno-electrode(acTnI/Cys/AuNC/APTMS/ITO).

S5. Effect of incubation time on immuno response

The time of interaction of immuno-eletrode BSA/acTnI/cys/AuNC/APTMS/ITO with cTnI i.e., incubation time was monitored at 50ng/ml concentration of cTnI by recording the increase in current at this concentration (**Figure S5**). It was observed that maximum rise in anodic peak current was recorded after 20 m of incubation. Further, an increase in time of incubation, the decrease in current was observed, which signifies that the binding is not permanent and desorption occurs after a particular time. Hence, 20m was used as optimised time for further study.

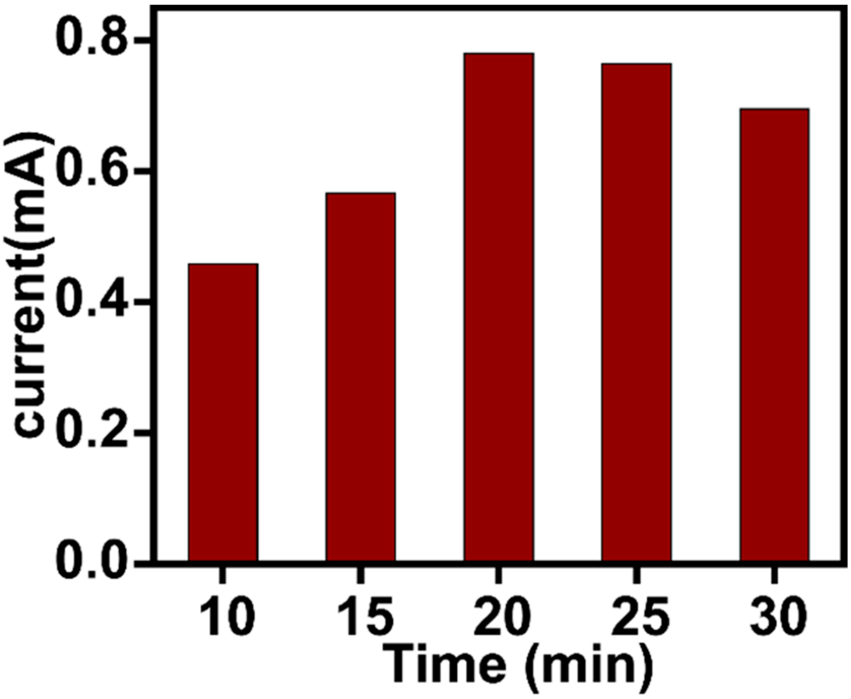

Figure S5. Effect of incubation time (from 10m to 30m) on the response of immuno- electrode towards cTnI..

S6. Effect of operational pH on the immuno-response of the fabricated immuno-electrode

The immuno-response of the proposed electrode BSA/acTnI/Cys/AuNC/APTMS/ITO was recorded by varying operational pH from 6.5 to 7.8 (**Figure S6**). It was found that maximum increase in response current is obtained at pH 7.4 and further increasing the pH leads to decrease in the current as at very high pH, the desorption of the analyte occurs.

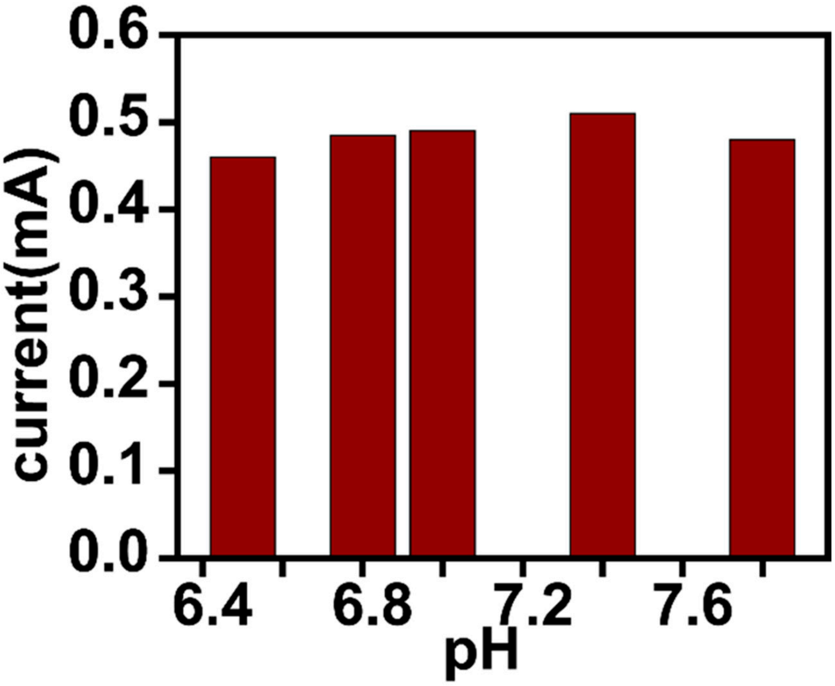

Figure S6. Effect of operational pH (variation from 6.5 to 7.8) on the immuno-response of acTnI/Cys/AuNC/APTMS/ITO electrode towards cTnI sensing.

S7. Interference study of the proposed Immuno-electrode

A interference study was performed for detection of cardiac troponin I 25ng/ml conc. in presence of similar concentration of isomeric form of cardiac troponin (cTnT) and BSA respectively. It was observed that the percentage interference by BSA (6%, RSD: 3.26%) and cTnT (14%, RSD: 6.5%) was minimum and within the permissible limit(<15%).

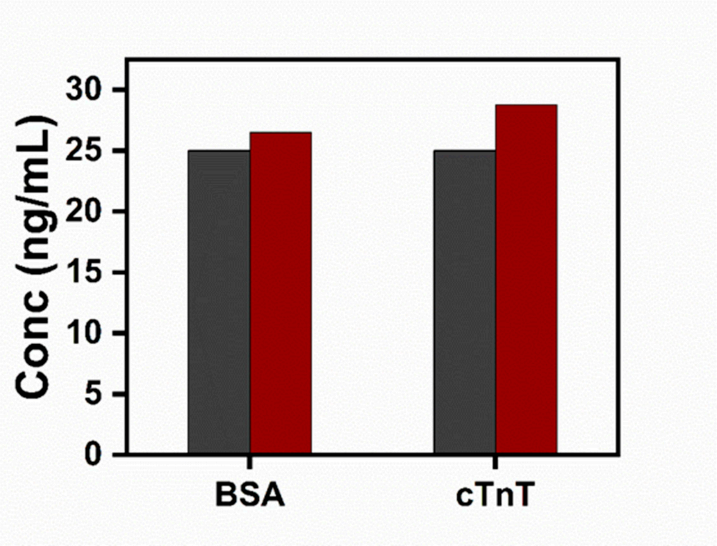

Figure S7. Study of interference by presence of other isomeric form (cardiac troponin T cTnT) and BSA on detection of cardiac troponin I (cTnI) for 25 ng/ml concentration.
